# NetTopoBFT: Network Topology-Aware Byzantine Fault Tolerance for High-Coverage Consortium Blockchains

**DOI:** 10.3390/e27111088

**Published:** 2025-10-22

**Authors:** Runyu Chen, Rangang Zhu, Lunwen Wang

**Affiliations:** College of Electronic Engineering, National University of Defense Technology, Hefei 230037, China; chenrunyu17@nudt.edu.cn (R.C.); zhurangang17@nudt.edu.cn (R.Z.)

**Keywords:** blockchain, Byzantine Fault Tolerance, network topology awareness, node reputation model, verifiable random function

## Abstract

The Practical Byzantine Fault Tolerance (PBFT) algorithm, while fundamental to consortium blockchains, suffers from performance degradation and vulnerability of leader nodes in large-scale scenarios. Existing improvements often prioritize performance while lacking systematic consideration of the structural characteristics of the nodes and network coverage. In this paper, a new network topology-aware Byzantine fault-tolerant algorithm NetTopoBFT is proposed for the supply chain and other application scenarios that require strict transaction finality but moderate throughput. Firstly, it innovatively combines the weighted signed network with the consortium chain, constructs a two-layer Bayesian smoothing node evaluation model, and evaluates the nodes through the two-dimensional evaluation of ‘behavioral reputation plus structural importance’. Then, to reduce the risk of being attacked, it uses Verifiable Random Function (VRF) to decide the leader. Furthermore, it uses a duplicate coverage-driven waitlisting mechanism to enhance the robustness and connectivity of the system. Theoretical analysis and experiment results show that NetTopoBFT significantly improves the quality of consensus nodes under the premise of guaranteeing decentralization, realizes the simultaneous optimization of communication overhead, security and network coverage. It provides a new idea for designing consensus mechanism of consortium blockchains.

## 1. Introduction

As an important branch of blockchain technology, consortium blockchain has demonstrated broad application potential in fields such as finance [1] and supply chain [2] due to its controllable access mechanisms, efficient transaction processing capabilities, and customizable privacy policies.

Consensus mechanism [3,4,5] is an indispensable core component of blockchain. It serves as the fundamental guarantee for maintaining data consistency and significantly influence the system’s security, decentralization level, scalability, and other performance metrics. As the core algorithm of consortium blockchains, Practical Byzantine Fault Tolerance (PBFT) [6] not only fully addresses Byzantine faults but also enables efficient, secure consensus and strong consistency guarantees with a limited number of nodes. However, as the scale of nodes in application scenarios increases, PBFT still faces two issues: first, performance degradation in large-scale node environments—as the number of nodes increases, the communication complexity of PBFT grows exponentially, leading to a significant decline in system performance [7,8,9]; second, leader node failure issues—PBFT’s static leader node switching strategy makes leader nodes more susceptible to malicious attacks [10,11].

The FLP impossibility theorem [12] and the CAP theorem [13] reveal that there is no consensus algorithm that can be applied to any scenario, and that although the design of any consensus algorithm allows for adjustable CAP balance, it cannot simultaneously satisfy consistency, availability, and partition tolerance. Therefore, existing improvements to the PBFT algorithm are mainly based on the actual needs of specific scenarios in which consortium chains are used, mainly focus on the following three aspects [14]:

- Towards more efficient BFT consensus: Efficiency of replication is the highest priority for most BFT applications, including permissioned blockchain platforms such as HyperLedger and CCF [15]. After PBFT pioneered a practical solution for BFT consensus, numerous approaches have proposed extensions and optimizations to improve system performance. Among these, leader-based BFT algorithms have gained favor due to their efficient coordination in the consensus processes during normal operation.
- Towards more robust BFT consensus: While efficient BFT algorithms achieve high performance under normal operation, they may have sacrificed robustness and become vulnerable under a diverse attack vector. The second category comprises BFT algorithms that prioritize robustness and security against various attacks and malicious behavior. Key aspects under this category include defending against various attacks, penalizing misbehavior, and improving fairness in ordering transactions.
- Towards more available BFT consensus: These studies prioritize availability and system responsiveness under different network conditions. On the one hand, they have developed asynchronous BFT protocols that are capable of operating in scenarios with varying levels of synchrony. On the other hand, considering many leader-based algorithms are susceptible to single points of failure, particularly when targeting the primary server. In such cases, the system must first detect a primary failure and then invoke a view-change protocol to select a new primary, they have developed leaderless BFT protocols.

From Table 1, we can find that most existing algorithms focus on improving performance during the design process and do not prioritize security optimization as a core objective. Instead, they may introduce new security risks. For example, Hotstuff reduces the number of interactions rounds by pipelining the three-phase commit process, but its node election relies on randomness, which may result in high-connectivity nodes being frequently selected. Attackers can monitor the network topology over the long term, predict and penetrate frequently selected nodes, and gain continuous control over the consensus process.

Specifically, the security of a consortium blockchain highly depends on the reliability and reasonable distribution of consensus nodes. On the one hand, attackers may carry out Byzantine attacks by infiltrating or partially controlling nodes, thereby disrupting system execution. On the other hand, if consensus nodes are too concentrated in the physical network topology (e.g., in terms of geographical regions, communication hubs, or logical connectivity), attackers can launch low-cost attacks by controlling a few key nodes (e.g., nodes with high betweenness centrality), leading to the failure of network-wide consensus. Ref. [28] points out that synchronous networks (such as consortium blockchains) require high coverage to ensure liveness. Ref. [31] experimentally demonstrates that in a 100-node consortium blockchain, latency grows exponentially when the neighbor coverage of PBFT is less than 60%, [32] notes that the sorting service based on BFT-SMaRt in Hyperledger Fabric requires at least two-thirds node coverage (66.7%) to ensure fault tolerance.

In existing research, [33] first determines the local reputation of nodes based on the heterogeneity of information transmitted between adjacent nodes of the same type and local information, given the heterogeneity of battlefield IoT nodes. It then uses the EigenTrust model to aggregate global reputation, forming a reputation model for the nodes. Ref. [34] proposes a reputation model for vehicle nodes based on dynamic grouping. This model combines vehicle reputation with geographical location information, grouping vehicles with good reputation and proximity to roadside units to enhance consensus efficiency. RBFT [35] introduces redundant backup nodes to enhance fault tolerance, with backup node selection based on static configuration or online status. These algorithms address the impact of node structure or network characteristics on consensus from different perspectives, but they do not systematically incorporate the structural characteristics of consortium chain nodes into the design of consensus algorithms.

Based on the above considerations, this paper focuses on two core objectives: security enhancement and network coverage optimization. For application scenarios such as supply chains, which have strict requirements for transaction finality but moderate throughput demands, we propose a novel network topology-aware Byzantine fault-tolerant algorithm called NetTopoBFT. This algorithm deeply integrates the structural characteristics of consortium chains with dynamic network coverage metrics, aiming to address the balance between security and performance in high-coverage scenarios under existing consensus mechanisms. The main innovations are as follows:

- By leveraging the node structure characteristics of a consortium blockchain, this study establishes a connection between weighted signed networks and consortium blockchains for the first time. Based on the weighted signed network, a communication model for the system is established, and then a two-layer Bayesian smoothing node evaluation model is designed. This model utilizes the node’s original reputation and the system’s global reputation as the two layers of smoothing to ultimately derive a unique global reputation for the node. This method not only can enhance the fault tolerance of the consortium blockchain but also provides a more comprehensive assessment of a node’s actual value through a dual-dimensional evaluation of ‘behavioral reputation + structural importance’.
- A new consensus protocol and view-change protocol is designed. We first optimize the commit phase based on the PBFT three-phase consensus protocol and design an autonomous decision-making cross-verification mechanism based on neighbor node reputation to reduce the communication complexity of the consensus protocol, Meanwhile, we use a duplicate coverage-driven waitlisting mechanism to ensure network consistency before and after the view-change protocol.
- Theoretical analysis and experimental verification of the proposed algorithm from multiple aspects, including node selection, network communication overhead, network coverage, and network security are conducted. The results show that the algorithm can not only effectively select high-quality consensus nodes based on the structural characteristics of nodes in the consortium chain while ensuring a certain degree of decentralization, but also, to our knowledge, it is the first consortium chain BFT algorithm that achieves simultaneous optimization of communication overhead and security without affecting network coverage.

The remainder of this paper is organized as follows: Section 2 introduces the system model. Section 3 discusses the design of the NetTopoBFT algorithm and Section 4 presents the theoretical analysis. Section 5 evaluates the NetTopoBFT by conductive simulations and Section 6 concludes the whole work.

## 2. System Model

In this section, we will introduce system models, which are divided into the network model and threat model. In the system model, we introduce a weighted signed network as the structural model of the consortium chain and, based on this, define the maximum number of communicable neighbors for nodes, the actual set of communication objects, and the communication selection mechanism for nodes under different circumstances. In the threat model, we will combine the node structure characteristics of the consortium chain to introduce two types of threat attacks that exist in the environment and target different node types.

### 2.1. Network Model

A Weighted Signed Network (WSN) is a complex network model where the edges between nodes not only have weights representing connection strength or frequency but also carry signs (typically ‘+’ or ‘−’), indicating positive (trust, cooperation) or negative (competition, conflict) relationships. This network model can simultaneously capture the intensity and polarity of interactions between nodes, making it widely applied in fields such as social networks and an important tool for understanding the dynamic multi-dimensional relationships in real-world systems.

In the context of consortium blockchains, this network model demonstrates unique applicability: on one hand, consortium blockchain nodes exhibit complex cooperative and competitive relationships, and the weighted signed network can precisely capture the trust, conflict, and interaction intensity between nodes through weighted edges with signs; on the other hand, the access mechanisms and consensus processes of consortium blockchains can be optimized based on the structural characteristics of the signed network. Additionally, by analyzing the cumulative distribution of weights in the network, it is possible to design anti-Sybil attack schemes based on reputation mechanisms to incentivize long-term cooperative behavior.

**Figure 1:**
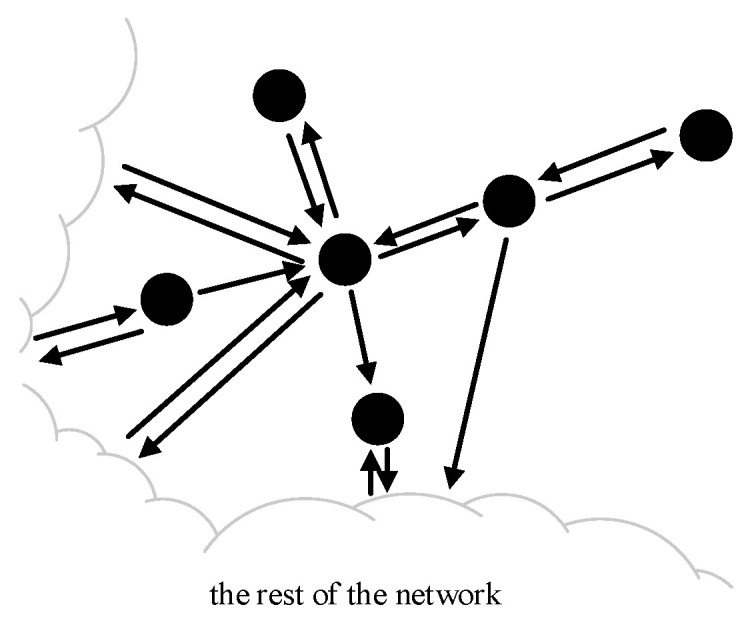
A fragment of network composed of 6 nodes.

Based on this, we abstract the consortium blockchain into a weighted signature network graph structure model that describes the complex interaction relationships among multiple entities. Let $G=\left(V,E,W\right)$ denote the weighted signed network, where V is the set of nodes representing independent entities (such as individuals, devices, or abstract units), $n=\left|V\right|$, E⊆V×V represents the set of edges, indicating the connections between entities in the network, W:E→[−Δ , Δ] is the mapping that assigns a value −Δ~Δ to each edge. W(i,j) can be described as the trust level of node i towards node j, where the symbol denotes the positive or negative nature of the relationship, and the numerical value (weight) represents the strength or trust level of the relationship. $N(i)=\left\{j|\;\left(i,j\right)\in E\right\}$ is the set of adjacent nodes of node i, and $d_{i}=\left|N(i)\right|$ denotes the degree of node i. A concrete example of a weighted signed network fragment is illustrated in Figure 1, which depicts the interaction relationships among 6 nodes.

In the above network model, the communication capabilities of nodes are limited by their resource constraints. This limitation directly affects the neighbor selection strategy of nodes in the consensus process, which in turn determines the communication efficiency and security of the entire network. To this end, we need to formally describe the communication behavior of nodes under resource constraints, specifically through the following three core definitions:

**Definition** **1.** ***Maximum number of communicable neighbors.** The maximum number of communicable neighbors represents the maximum number of neighbors that a single node can establish communication connections with simultaneously under the constraints of global resource parameters. It represents the upper limit of a node’s communication capacity under resource constraints.*

(1) $$c_{i}=\left\lceil k\cdot d_{i}\right\rceil$$

*where *k* is global resource constraint parameter,* k∈(0,1].

**Definition** **2.** ***Actual communication object set.** The actual communication object set refers to the set of neighboring nodes that a node ultimately selects for communication under resource constraints. This set is a subset of the node’s adjacent node set that meets the communication conditions.* $S_{i}^{\left(t\right)}\subseteq N(v_{i})$ *which satisfy* $\left|S_{i}^{\left(t\right)}\right|\subseteq c_{i},\;\forall t$.

**Definition** **3.** ***Node communication decision function.** The node communication decision function is a function used to select the actual communication object strategy. This function determines how nodes filter out the final communication object combination from the set of neighboring nodes. The selection is typically based on trustworthiness, strength of relationship, or other strategic factors. This paper adopts the following node communication decision function* $S_{i}^{\left(t\right)}=f(N(i),\;R,\;\tau )$.

(2) $$f(N(i,\;R,\;\tau )=\left\{\begin{array}{ll} Top\_\tau (R,\;N(i),\;\{j|R(j)>0\})\; & \left|\left\{j\in N(i)|R(j)>0\right\}\right|\geq \tau \\ \left\{j\in N(i)|R(j)>0\right\} & \left|\left\{j\in N(i)|R(j)>0\right\}\right|<\tau ,\;\zeta =0\\ \left\{j\in N(i)|\right\} & \left|\left\{j\in N(i)|R(j)>0\right\}\right|<\tau ,\;\zeta =1 \end{array}\right.$$

*where* Top_τ(R, N(i), {j|R(j)>0}) *represents the nodes* R(j)>0 *filtered from* N(i) *and sorted in descending order, taking the top* τ *nodes,* ζ *is a random variable,* $\zeta \in \left\{0,1\right\}$*,* P(ζ=0)=P(ζ=1)=0.5*;* R(j) *represents the credibility value of node* j*, and the specific calculation method will be discussed in Section 3.1.*

### 2.2. Threat Model

The primary security threats faced by blockchain systems include Sybil attacks, DDoS attacks, and others, with the effectiveness of such attacks fundamentally dependent on whether the attacker can obtain majority control within the system. However, in a consortium blockchain architecture based on NetTopoBFT, the selection mechanism for consensus nodes is directly linked to node reputation—this reputation value is calculated based on the explicit and dynamically manageable node structure characteristics of the consortium blockchain. Based on the research findings on consortium blockchain security threats in [36,37], the system threat model can be summarized into the following two core dimensions from the perspective of node topology structure:

(1) Edge node penetration attacks

The attack path of ‘penetrating from the edge to the core’ is not only the attacker’s preferred strategy, but its success rate is also significantly higher than other attack methods. Consortium blockchain networks typically exhibit centralized or hierarchical characteristics: core nodes have high connectivity but are scarce in number, while edge nodes are numerous but have low connectivity. This topological structure naturally forms penetration channels from peripheral weak points to internal core areas. Attackers control peripheral edge nodes in the consortium blockchain, leverage the system’s trust propagation mechanism to achieve lateral movement, gradually breach the internal network, and ultimately complete penetration of the core area.

(2) Targeted attacks on core nodes

Attackers adopt a precision strike strategy, directly targeting key roles in the consortium blockchain’s consensus protocol for corruption. By manipulating the consensus process through malicious proposals, fraudulent voting, and other means, they not only cause chain state splits and system trust collapse but also gain a ‘god’s-eye view’ or absolute control over the entire network. Compared to peripheral node penetration, the scope and severity of damage from such attacks, once successful, will expand exponentially.

In terms of security, we conducted a theoretical analysis of the aforementioned threat model in Section 4.1.

## 3. The Design of NetTopoBFT

This section describes the detailed design of the NetTopoBFT algorithm. We first provide an overview of the algorithm, then explain the algorithm components in detail. Table 2 lists the parameter symbols used in the system design description.

### 3.1. Framework Overview

**Figure 2:**
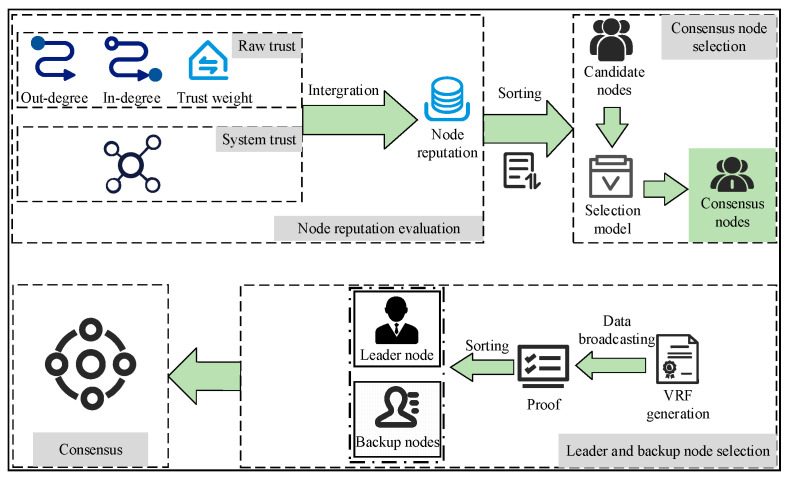
The workflow of NetTopoBFT.

Based on the overview of the system model in Section 2, Figure 2 briefly introduces the workflow of NetTopoBFT, which can be roughly divided into four parts.

- Node reputation value calculation: A two-dimensional evaluation of ‘behavioral reputation + structural importance’ is adopted, and a two-layer Bayesian smoothing is used to calculate the unique global reputation value of each node.
- Consensus Node Selection: Based on the calculated global reputation value, nodes are further screened according to their structural characteristics to select eligible nodes as consensus nodes.
- Leader Node and Its Backup Node Election: A verifiable random function is used to randomly select nodes from the consensus nodes as leader nodes, and corresponding backup nodes are selected based on the node coverage repetition rate metric.
- Blockchain consensus phase: After the leader node and its backup nodes are determined, the system enters a consensus process based on an improved PBFT. This phase introduces a dynamic weighting voting mechanism, using the node’s global reputation value as the voting weight. Concurrently, real-time coverage detection of backup nodes is integrated. If the leader node fails, the corresponding backup node can quickly assume proposal authority, ensuring communication efficiency optimization and topological structure stability for the consensus process.

### 3.2. Node Reputation Value Calculation

Node reputation serves as a core metric for evaluating a node’s trustworthy behavior and contributions to the network, directly impacting the rationality of consensus elections, the security of the consensus process, and the reliability of consensus outcomes. NetTopoBFT employs a dual-dimensional evaluation framework combining ‘behavioral reputation’ and ‘structural importance’, generating a unique global reputation value for each node through multi-stage calculations. First, it extracts the node’s baseline reputation (raw reputation) based on interaction behavior within the weighted signed network. Then, it combines the network-wide connection characteristics to construct a benchmark reference value (global average reputation) and uses double Bayesian smoothing to obtain the node’s global reputation value.

#### 3.2.1. Node’s Original Smooth Reputation

Node degree refers to the total number of edges directly connected to a node and is a core topological indicator for measuring the local importance of a network. In undirected networks, degree typically represents the scale of neighboring nodes—nodes with higher degrees often serve as network hubs or central nodes, possessing stronger communication capabilities and reliable connection reserves. Therefore, they should theoretically be prioritized as consensus nodes to ensure network efficiency and stability. Given the directed communication characteristics of consortium blockchains, the in-degree and out-degree must be used to precisely characterize the communication direction and functional roles between nodes:

In-degree: The number of directed edges pointing to the node, reflecting its passive influence as an information recipient. The in-degree credibility weight represents the level of trust other nodes have in the target node, reflecting the scale of its active trustworthiness.

Out-degree: The number of directed edges originating from the node, representing its ability to actively connect to other nodes, reflecting the control power and network resource dominance of the information initiator. Out-degree credibility weight is used to assess the node’s trust level toward the recipient, directly influencing its information broadcasting strategy.

Correspondingly, we can obtain the node’s original in-degree credibility and original out-degree credibility:

(3) $$R_{in}\prime (i)=\frac{\sum_{j\in N(i)}W(j,i)}{deg\_in(i)}$$

(4) $$R_{out}\prime (i)=\frac{\sum_{j\in N(i)}W(i,j)}{deg\_out(i)}$$

To avoid situations where credibility assessments are distorted due to extreme in-degree or out-degree values of nodes, such as establishing a small number of high-quality connections to mask overall low participation, we introduce a first-layer smoothing calculation to obtain the original smoothed in-degree credibility and original smoothed out-degree credibility of nodes:

(5) $$R_{in}(i)=\frac{\sum_{j\in N(i)}W(j,i)}{deg\_in(i)+\mu }$$

(6) $$R_{out}(i)=\frac{\sum_{j\in N(i)}W(i,j)}{deg\_out(i)+\upsilon }$$

where μ,υ are the smoothing parameters.

(7) $$\mu =\frac{1}{n}\sum_{i\in V}deg\_in(i)$$

(8) $$\upsilon =\frac{1}{n}\sum_{i\in V}deg\_out(i)$$

Here, parameters μ,υ are used to suppress extreme values of low in-degree and low out-degree for nodes, balancing the bias caused by network structural heterogeneity and making the credibility value more focused on the actual interaction quality of nodes rather than simply the number of connections. Specifically, when deg_in(i)≪μ or deg_out(i)≪υ, the corresponding value $R_{in}(i),R_{out}(i)$ is proportionally reduced, thereby minimizing the interference of low-activity nodes on reputation assessment; conversely, in other cases, the suppression term gradually approaches 0 as the number of connections increases, ensuring that the reputation assessment of highly connected nodes remains unaffected.

Finally, the original smoothed reputation of the node can be obtained.

(9) $$raw\_r(i)=\frac{1}{2}\times R_{in}(i)+\frac{1}{2}\times R_{out}(i)$$

#### 3.2.2. Global Average Reputation

The global average reputation is a benchmark metric used to measure the average reputation level of all nodes within the entire system. It is defined as the arithmetic mean of the original reputation values of all nodes in the network.

(10) $$global\_avg=\frac{\sum_{i\in V}\sum_{j\in V}W(i,j)}{\left|E\right|}$$

The core purpose of calculating global average reputation is to eliminate the impact of local node behavior biases on reputation assessment. In distributed networks, node connection distributions and interaction behaviors may vary significantly due to differences in topological location or role (e.g., edge nodes vs. core nodes). By introducing global average reputation as a benchmark, the system can compare individual reputations with overall levels to identify abnormal nodes that deviate significantly from the baseline (whether too high or too low), thereby enhancing the robustness and fairness of reputation assessment.

From the perspective of the node evaluation model, global average credibility serves as a critical bridge connecting individual behavior with system-level decision-making. It provides the necessary benchmark parameters for double Bayesian smoothing, ensuring that the final global credibility value reflects both the node’s own behavioral characteristics and aligns with the system’s overall state.

#### 3.2.3. Node’s Global Reputation

The global reputation is constructed by combining the original smoothed reputation of the node with the system-wide average reputation, creating a comprehensive evaluation metric that integrates individual behavioral characteristics with a system-wide perspective. The calculation formula is as follows:

(11) $$R(i)=\left\{\begin{array}{ll} \frac{d(i)}{d(i)+\beta }\;\times \;global\_avg+(\frac{\beta }{d(i)+\beta })\;\times raw\_r(i) & global\_avg\geq \bar{raw\_r(i)}\\ \frac{d(i)}{d(i)+\beta }\;\times \;raw\_r(i)+(\frac{\beta }{d(i)+\beta })\;\times global\_avg & global\_avg\leq \bar{raw\_r(i)} \end{array}\right.$$

where $\bar{raw\_r(i)}$ represents the average of the original smoothed reputation for all nodes, and β is the weight used to control the global prior.

(12) $$\bar{raw\_r(i)}=\frac{1}{n}\sum_{i\in V}raw\_r(i)$$

(13) $$\beta =\frac{\left|E\right|}{\left|V\right|}$$

R(i) serves as the second layer of smoothing, using parameters β to suppress nodes with excessively low total connection counts. When $global\_avg\geq \bar{raw\_r(i)}$, as the total number of connections for a node approaches positive infinity, the global prior weight approaches 1, allowing the global reputation of the corresponding node to approach the global benchmark value infinitely. Conversely, as the total number of connections approaches 0, the single-connection weight approaches 1, at which point the node’s reputation depends entirely on its original smoothed value. Conversely, when $global\_avg\leq \bar{raw\_r(i)}$, as the total number of connections for a node approaches 0, the global prior weight approaches 1, causing the global reputation of the corresponding node to approach the global benchmark value. As the total number of connections for a node approaches positive infinity, the single-connection weight approaches 1, enabling the global reputation of the corresponding node to approach its original smoothed value infinitely, thereby further enhancing the robustness and fairness of reputation assessment.

Additionally, it is important to note that we did not assign weights to node connectivity as an independent metric. This is because, in real-world networks, the frequency distribution of node connectivity tends to be right-skewed, with a small number of nodes exhibiting extremely high connectivity. Taking the Bitcoin OTC network as an example, the network contains 5881 nodes, with over 75% of nodes having connectivity distributed around the median of 4. However, there is also a node with connectivity of 795. If this were treated as an independent metric, the final calculation results would exhibit significant bias. The algorithm we employ effectively addresses this issue.

### 3.3. Consensus Node Selection

The election of consensus nodes is a critical component in ensuring the secure and efficient operation of the system. Consensus nodes not only bear the core responsibilities of transaction verification and block consensus, but the quality of their selection directly impacts the overall performance and security thresholds of the entire network. This paper proposes a consensus node election mechanism based on node reputation assessment and topological characteristics. This mechanism is designed from two aspects: node qualification screening and node number determination. It not only ensures the basic communication capabilities and bidirectional interaction credibility of candidate nodes but also achieves dynamic optimization of the consensus group size.

First, we need to discuss how to select nodes eligible to become consensus nodes based on their reputation. To further build a consensus node set that combines high credibility and strong connectivity, and to prevent the emergence of ‘fake low-connectivity high-quality nodes’, we establish the following two core screening principles based on the node evaluation model proposed in Section 3.1:

P1: deg_in(i),deg_out(i) > 0

It reveals that connectivity is the foundation of consensus. On the one hand, it meets the requirements of network topology completeness. Nodes with only in-degree or out-degree (such as ‘unidirectional nodes’ that only receive or send information) may cause network communication links to break, increasing the risk of delays in consensus message transmission. On the other hand, it meets the requirement for interaction symmetry. The consensus process requires nodes to be able to both receive and verify proposals, as well as actively initiate votes or synchronization requests. Bidirectional connections are the fundamental condition for achieving symmetric interaction.

$$P2:\;R_{In}(i)\;,\;R_{Out}(i)\;>\;0$$

To ensure that nodes demonstrate a certain degree of positivity and credibility in terms of information inflow and outflow, the sum of the in-degree credibility value and out-degree credibility value of the selected nodes must be greater than 0. It reveals the two-way balance of credibility. On the one hand, it is used to prevent ‘free riding’ behavior and reduce the implicit strategic speculative risk. On the other hand, the dual thresholds of entry and exit credibility can screen out nodes that are active in both information reception and dissemination, avoiding consensus imbalances caused by unilateral behavior.

For convenience of description, we denote the set of nodes that satisfy the above conditions as N′, and the number of nodes in the set as n′.

Next, we need to discuss how to further determine the number of consensus nodes in the network. Considering that the communication complexity of the PBFT algorithm is $O(n^{2})$, an increase in the number of nodes will cause the communication complexity of the system to grow exponentially. To further reduce the communication complexity of the PBFT algorithm in large-scale consortium blockchains, this paper proposes a dynamic proportional adjustment mechanism.

(14) $$c=\left\lfloor \eta \sqrt{n\prime }\right\rfloor$$

where η is the adjustment coefficient used to balance system security and communication efficiency.

Specifically, the above design limits the number of consensus nodes c to the order of magnitude $\sqrt{n}$, theoretically reducing communication overhead from $O(n^{2})$ to O(n). This significantly alleviates the message flood problem in large-scale networks.

### 3.4. Leader Node and Its Backup Node Election

In the PBFT algorithm, the traditional leader node election strategy uses a rotating ‘dealer’ mechanism. While this deterministic selection method simplifies the election process, it introduces significant security risks: attackers can predict the identity of the leader node and launch targeted attacks (such as DoS attacks), forcing the system to frequently trigger view changes (View Change). This not only increases communication overhead but also causes a sharp decline in system performance. To address this issue, this paper uses a leader node random election model based on verifiable random functions (VRF) [38], which significantly increases the difficulty for attackers to predict while ensuring fairness. Additionally, it combines a node coverage repetition rate metric to optimize the selection strategy for candidate nodes, thereby enhancing the system’s fault tolerance and dynamic adaptability.

#### 3.4.1. VRF-Based Random Election Mechanism for Leader Nodes

Similar to [39], this model introduces VRF into consensus nodes and uses cryptographic to achieve unpredictable election. Its core process consists of the following three stages:

Phase 1: VRF computation and proof generation

Each node in the consensus node set uses its own private key sk and the current consensus round number λ to calculate the local output result and proof, and generates a timestamp $t_{i}$ for the above result:

(15) $$result=VRF_{Hash}(sk_{i},\lambda )$$

(16) $$proof=VRF_{\mathrm{Pr}oof}(sk_{i},\lambda )$$

Phase 2: Distributed verification and sorting

Node submit its result and proof to other nodes. Upon receiving the corresponding result and proof, the relevant nodes first use formula $result=VRF_{P2H}(proof)$ to verify the validity of the above output. If it is true, $True/False=VRF_{verify}(pk_{i},\lambda ,proof)$ is calculated, where True indicates that the verification is successful and False indicates that the verification is unsuccessful.

Phase 3: Backup node selection strategy based on node coverage redundancy rate

All validated nodes are sorted based on their own result, and the node with the largest result is selected as the master node for the current round. If multiple nodes simultaneously produce the maximum VRF output, the system will select the node with the smallest $t_{i}$ as the leader node.

The unpredictability and verifiability of VRF ensure that attackers cannot infer the identity of future leader nodes based on historical data, while any forged outputs can be quickly verified and rejected by other nodes, eliminating the possibility of malicious nodes impersonating leaders.

#### 3.4.2. Candidate Node Selection Strategy Based on Node Coverage Overlap Ratio

When leader node j is found to be down or exhibiting malicious behavior, the system must quickly elect a backup node j′ to maintain consensus stability. This paper proposes using the node coverage overlap ratio (NCOR) as the basis for selecting candidate nodes, with the calculation formula shown below.

(17) $$NCOR_{jj\prime }=\frac{\left|N_{j}\cap N_{j\prime }\right|}{\left|N_{j}\cup N_{j\prime }\right|}$$

If multiple nodes have the same NCOR, select the node with the highest R.

The use of node coverage repeat rate as the basis for selecting candidate nodes primarily considers the following two factors: First, communication efficiency optimization—high NCOR nodes share more neighbors with the original master node, enabling rapid reconstruction of consensus message propagation paths and reducing network convergence time; second, stability assurance—nodes with high adjacency repetition rates are typically located at the core of the network (e.g., data center backbone nodes), possessing stronger bandwidth resources and fault tolerance capabilities, enabling them to adapt to dynamic network environment changes.

### 3.5. Blockchain Consensus Phase

After the election of the leader node and candidate nodes is completed, this phase improves the existing three-phase consensus process of PBFT through node selective verification and a weight-based decision-making mechanism. The specific process is as follows:

Phase 1: Pre-prepare phase

The master node packages a new block $B_{t}$ and distributes it to all consensus nodes via a network broadcast. The message format is ${\mathrm{Pre}-\mathrm{prepare}}_{L}(v,A,B_{t})$, where v is the view number and A is the request summary.

Phase 2: Prepare phase

Different from PBFT, this phase introduces a selective verification mechanism based on node’s global reputation. The decision logic for consensus nodes is as follows:

(1) Direct decision branch: To reduce the additional communication complexity introduced by node cross-validation, any node may choose the direct decision branch based on its own circumstances. Specifically, there are the following scenarios: For malicious node i, they are highly likely to choose direct decision branch to seek to circumvent the cross-verification process to transmit false information or disrupt the normal operation of the system’s consensus protocol. For honest node i, they have the following two possible choices for the direct decision branch: one is R(i)≥max(R(j),j∈N(i)), that is the node’s own global reputation is greater than any of its neighboring nodes so that there is no need to require other nodes’ verification, the other is when ∃R(j)≥R(i),j∈N(i), honest nodes still have a 50% probability of selecting the direct decision branch. If node i chooses direct local verification, it directly generates ${\mathrm{Commit}-1}_{i}(B_{t},approval/against)$ and sends to the master node, where approval represents approval, against represents opposition, and the node will exit the subsequent consensus process. If node i chooses direct local verification, it directly generates ${\mathrm{Commit}-1}_{i}(B_{t},approval/against)$ and sends to the master node, where approval represents approval, against represents opposition, and the node will exit the subsequent consensus process.

(2) Cross-verification branch: In other scenarios, nodes typically select the cross-verification branch to ensure the authenticity and consistency of information transmission. If node i chooses further verification, it selects nodes with higher reputation values from the neighbor set N(i) to form a subset N′(i)⊆N(i)∩C, and sends a message ${\mathrm{Prepare}}_{i\to j}(v,A,B_{t})$ to ∀j∈N′(i).

Therefore, for direct decision branches, the final decisions may carry a certain degree of risks. To minimize the impact of this risk on the final decision, we have capped the proportion of this component in the calculation of the final decision weighting.

Phase 3: Commit phase

(1) Message generation: Nodes that did not make a direct decision in Phase 2 generate ${\mathrm{Commit}-2}_{i}(B_{t},approval/against)$ based on the received Prepare message set verification.

(2) Weight Calculation: Each decision result is recorded on the blockchain. The master node aggregates all Commit-1 and Commit-2 messages and calculates $W_{approval}$ and $W_{against}$ based on the global reputation of the decision-making node itself. If $W_{approval}>W_{against}$, $B_{t}$ is appended to the blockchain; otherwise, $B_{t}$ is deemed invalid, and L will no longer serve as a consensus node. The formula is as follows:

The election of consensus nodes is a critical component in ensuring the secure and efficient operation of the system. Consensus nodes not only bear the core responsibilities of transaction verification and block consensus, but the quality of their selection directly impacts the overall performance and security thresholds of the entire system.

(18) $$W_{approval}=0.5\times \sum_{i\in C}{\mathrm{Commit}-1}_{i}(B_{t},approval)\cdot R(i)+\sum_{i\in C}{\mathrm{Commit}-2}_{i}(B_{t},approval)\cdot R(i)$$

(19) $$W_{against}=-0.5\times \sum_{i\in C}{\mathrm{Commit}-1}_{i}(B_{t},against)\cdot R(i)-\sum_{i\in C}{\mathrm{Commit}-2}_{i}(B_{t},against)\cdot R(i)$$

**Figure 3:**
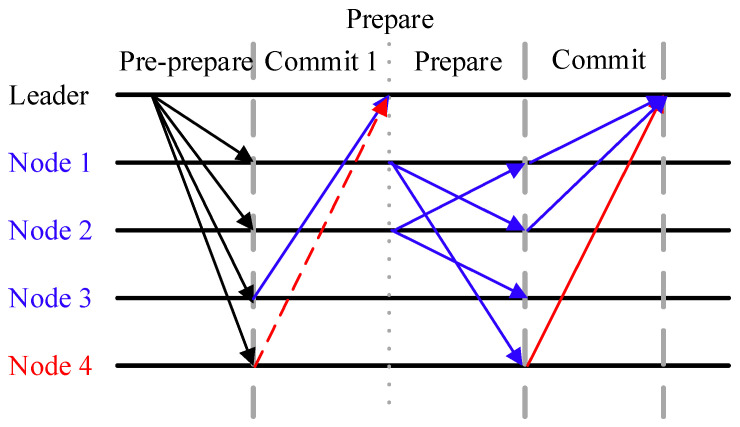
NetTopoBFT consensus diagram.

The specific consensus flowchart is shown in Figure 3, where blue represents honest nodes and their corresponding communication decisions, red represents malicious nodes and their corresponding communication decisions, and dashed lines represent optional decisions for nodes.

Compared to the fully connected pairwise communication model in the Practical Byzantine Fault Tolerance (PBFT) consensus mechanism, this algorithm eliminates redundant node interactions by optimizing the communication topology structure after selecting consensus nodes based on a reputation mechanism, significantly reducing the communication complexity of the consensus process. Additionally, this algorithm innovatively introduces a dynamic weight allocation mechanism, assigning differentiated weight coefficients to decisions submitted by different nodes based on the characteristics of the consensus phase. This enhances decision-making efficiency while strengthening the rationality verification of group decisions.

## 4. Theoretical Analysis

In this section, we mainly analyze the NetTopoBFT algorithm from the perspectives of security and activity. To analyze these two aspects intuitively and clearly demonstrate the advantages of the mechanism we propose, we compare our proposed algorithm with several widely used consensus mechanisms. The comparison results are shown in Table 3, which demonstrates the superiority of our algorithm.

### 4.1. Security Analysis

Based on the threat model outlined in Section 2, this section primarily analyzes the system’s security against two types of attacks: edge node penetration attacks and targeted attacks on core nodes.

#### 4.1.1. Edge Node Penetration Attacks

The most common approach involves attackers gaining control of edge nodes on the periphery of a consortium blockchain, exploiting the system’s trust propagation mechanism to achieve lateral movement, gradually penetrating into the internal network, and ultimately completing penetration into the core area. The algorithm we propose effectively resists such malicious behavior from two aspects to ensure system security. On one hand, we do not assign weight to node connectivity as a standalone metric, thereby avoiding the possibility of overly high connectivity playing a decisive role in the metric, which theoretically prevents the establishment of unnecessary connectivity between edge nodes. On the other hand, as shown in the table, if an attacker wishes to participate in consensus through edge node penetration attacks, they generally need to increase the connectivity of nodes to a certain scale. This action not only alters the original architecture of the consortium chain but is also marked as suspicious by the consortium chain’s regulatory system. Additionally, this behavior exceeds the communication capabilities of the nodes themselves to some extent.

#### 4.1.2. Targeted Attacks on Core Nodes

NetTopoBFT employs a VRF-based random leader node election mechanism and adopts weighted voting and majority decision principles during the consensus process to prevent this attack. On one hand, even if an attacker successfully carries out a targeted attack on a core node, they cannot accurately predict the ID of the leader node for this round of consensus, making it impossible to manipulate the consensus process. On the other hand, even if an attacker participates in the consensus and makes a false decision on the leader node’s block, due to the small differences in weights between nodes, it is impossible for a single malicious node to monopolize the weights. Taking the global reputation calculation results of nodes in the BitcoinOTC network as an example, among the top 150 nodes by reputation value, the maximum reputation value is 2.1257, the minimum reputation value is 1.9366, with a difference of approximately 0.15, and the variance of these 150 reputation values is 0.0008, which is almost negligible.

### 4.2. Liveness Analysis

(1) Fault tolerance: NetTopoBFT first uses a node reputation model to select consensus nodes, then uses the selected consensus nodes to complete the consensus process. Although the consensus process still uses PBFT’s fault tolerance capabilities, the actual algorithm’s fault tolerance capabilities exceed PBFT’s fault tolerance range.

(2) Liveness proof: This refers to a node’s ability to continuously and effectively process and confirm new operations, ensuring that all transactions are executed accurately without interruption. From the perspective of leader node replacement, replacing leader nodes based on node redundancy coverage ensures network connectivity and continuity before and after node replacement, enhancing system liveness while maintaining the original network information transmission efficiency. From the perspective of voting weights for nodes participating in consensus, although each node is assigned a corresponding voting weight, the overall differences in voting weights among nodes are negligible, thus avoiding issues where certain nodes monopolize voting power and undermine the enthusiasm of other nodes to participate in consensus.

(3) Data storage analysis: In the system model of the NetTopoBFT mechanism we are serving, we divide the nodes of the entire system into general nodes and consensus nodes. The latter have more storage space than the former to store blockchain data. However, as the duration of the blockchain-enabled IoT system increases, the number of transactions will significantly rise, leading to consensus nodes being unable to store all blockchain data. To save storage space, we can apply a social-based reduction method [40] to local blockchain data or upload part of the data to a cloud center for storage. Specifically, we can sort the transaction data of all nodes based on timestamps and then periodically upload some old data to nearby cloud centers for storage. This will be the focus of our future work.

## 5. Experimental Verification

In this section, we use Python to implement a simulation of NetTopoBFT on our computer. Our evaluation focuses on the following three aspects: first, the quality of the node selection model; second, communication complexity; and third, algorithm security. The algorithm security is primarily evaluated in terms of the randomness and unpredictability of leader node selection, as well as the security of NetTopoBFT based on the consensus process. The security of the consensus process is primarily verified by measuring the network coverage of the consensus (as illustrated in [32] using the Hyperledger Fabric platform as an example, a successful consensus requires at least two-thirds of the nodes to be covered to ensure fault tolerance).

### 5.1. Experimental Setup

All experiments were conducted on a Legion R9000P ARX8 laptop (Manufacturer: Lenovo Group Limited, Beijing, China) equipped with an AMD Ryzen 9 7945HX CPU (16 cores, 32 threads), 16 GB RAM. The programming language used was Python 3.9.13. Experiments simulated distributed nodes using Python’s multiprocessing library, with each node process allocated fixed CPU cores and memory resources to mimic the behavior of an independent physical node.

This paper selects four real-world weighted signed network datasets provided by [41], with their basic information shown in the table below. Bitcoin Alpha/OTC are trust networks of users who conduct transactions using Bitcoin on their respective platforms. Since Bitcoin users are anonymous, it is necessary to maintain records of user reputation to prevent transactions with fraudulent or high-risk users. This is also the first explicit weighted signed directed network available for research. REAnet is a dataset from Wikipedia’s election management system, where nodes represent Wikipedia members and edges represent votes. EpinionNet is a trust network derived from the review website Epinions.com, where trust relationships interact to form a trust network. Table 4 shows some key information of the four datasets.

In the current simulation, we have assumed an ideal local area network environment where communication delays between nodes are negligible and bandwidth is unrestricted. We will explicitly state this assumption in the revised version and clarify that it aims to isolate network physical layer disturbances, focusing on evaluating the performance of the algorithm’s logical layer.

### 5.2. Experimental Results

#### 5.2.1. Evaluation of Node Selection Models

In this section, we first summarize existing methods for calculating node reputation and then compare the consensus node selection results of NetTopoBFT with existing methods. For consortium blockchains, the selection of consensus nodes must take into account both node behavior and node structure. Specifically, nodes must not only have a certain degree of connectivity within the network but also be trusted by the majority of nodes.

Overall, BFT algorithms based on leaders design different node reputation calculation methods according to different application scenarios. Based on the calculation methods, they can be broadly categorized into the following four types: reputation calculation based on historical behavior performance, reputation calculation based on contribution, reputation calculation based on social relationship networks, and reputation calculation based on machine learning models. Further, these can be summarized into two approaches: reputation based on node behavior (Behavior-based Reputation) and reputation based on node structure (Structure-based Reputation).

Table 5 shows the node selection results for different datasets under different node ratios.

In Table 5, Q1 indicates that both the in-degree credibility and out-degree credibility symbols of a node are positive, signifying that the node possesses a high level of trustworthiness within the network. Additionally, Q1 indicates that the node’s connectivity is greater than or equal to the average connectivity of nodes in the network, indicating that the node has a certain level of connectivity within the network.

As shown in the table, in a consortium blockchain, compared to the other three types of algorithms, relying solely on node behavior credibility to establish a node credibility model can ensure that nodes have a high level of trustworthiness, but it cannot guarantee that nodes have a certain degree of connectivity within the network. If such nodes are selected to participate in consensus, it may lead to insufficient network coverage, making the entire system more vulnerable to attacks. When comparing the remaining three algorithms, it can be observed that the method proposed in this paper, which uses two-layer Bayesian smoothing to calculate node credibility, performs optimally overall, with stable performance and the ability to adapt well to networks of different scales. The performance of the node reputation model based solely on structural reputation is inferior. Although it may outperform the algorithm proposed in this paper under certain conditions, its performance exhibits significant variability. The algorithm based solely on Bayesian smoothing performs comparably to the other two algorithms when the network has a large number of nodes, but when the number of nodes is small, it fails to adequately filter out nodes with insufficient connectivity.

Further analysis of the reasons behind these results reveals that, for consortium blockchains, high behavioral credibility of nodes does not necessarily indicate high connectivity, as some nodes may collude to create a small number of high-quality connections to artificially boost their trustworthiness. However, better structural characteristics of nodes can to some extent indicate higher trustworthiness, which stems from both the consortium blockchain’s access mechanisms and relationship solidification, as well as the long-term accumulation of trust within the relationship network of the consortium blockchain. Therefore, it is essential to reasonably incorporate nodes’ structural characteristics into the evaluation criteria for node credibility.

#### 5.2.2. Communication Complexity

**Figure 4:**
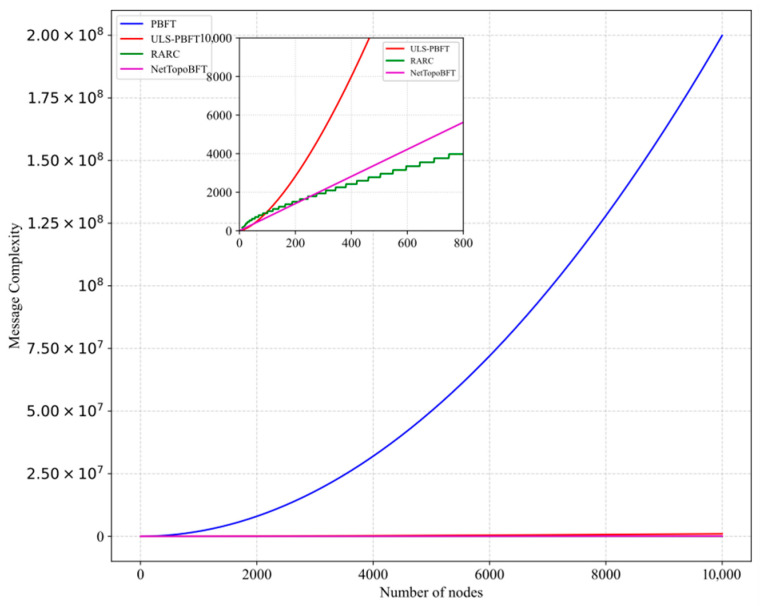
Comparison of communication complexity.

Figure 4 shows a comparison of communication complexity among different algorithms. PBFT has the highest communication complexity, approximately $O\left(n^{2}\right)$, as it relies on communication between nodes to achieve message confirmation. USL-PBFT [42] is designed for drone applications, dividing nodes into cluster head nodes and cluster internal nodes, which undergo separate consensus processes. Due to the need for information exchange between different clusters, its communication complexity is relatively high, approximately $O\left(n^{1.5}\right)$. RARC [39] incorporates genetic algorithms into the voting process, eliminating the need for nodes to reach consensus through mutual verification, resulting in the lowest communication complexity, approximately $O\left(n\right)$. Additionally, the algorithm sets different consensus processes for networks of varying scales, resulting in a staircase-like curve for communication complexity. The algorithm proposed in this paper, although it includes optional verification steps during the consensus process, significantly reduces the scope of node verification, resulting in communication complexity that can also be approximated as $O\left(n\right)$.

#### 5.2.3. Security Analysis

In this section, we will comprehensively analyze the security of the algorithm from four aspects: edge node penetration attack model; targeted attacks on core nodes model; the coverage of the consensus process network, and the duplicate coverage of the standby node network.

Regarding the first threat model, malicious nodes typically attack the system through two methods: firstly, different malicious nodes can multiple false high-trust-weight edges to boost a specific malicious node’s credibility; second, malicious nodes initially act honestly while concealing their identities, then launch attacks once they have accumulated sufficient trust weight. However, for NetTopoBFT, neither approach is feasible for the following reasons:

Take BitcoinOTC as an example, as demonstrated by the above analysis; malicious nodes often establish false high-trust weights among themselves to inflate their credibility within the system. Table 6 demonstrates the varying final reputation values achieved by malicious nodes in the BitcoinOTC dataset through establishing different numbers of fake high-trust-weight edges within the system. From Table 6, we discover that in NetTopoBFT comparing with the node with the largest reputation 2.25069, establishing fake high-trust-weight edges can indeed enhance the reputation of nodes; however, the number of high-trust-weight edges does not always correlate positively with reputation values.

However, in NetTopoBFT, a high reputation score merely signifies that a node qualifies to become a consensus node or leader node; it does not guarantee that it will be able to carry out a malicious attack. This is because although malicious nodes can artificially inflate their reputation by fabricating high-weight reputation edges, the maximum possible increase in overall reputation is approximately 0.14. This value translates to a weight in the consensus phase that is virtually negligible. Furthermore, considering the high cost for malicious nodes to fabricate high-trust edges, the proportion of malicious nodes during the consensus phase will be even lower in practice. Therefore, NetTopoBFT can effectively solve this threat model.

Regarding the second threat model, malicious nodes typically target nodes within the system that have a probability of becoming consensus nodes or leader nodes. In this scenario, Section 4.1.2 has already preliminarily demonstrated from the perspective of node reputation that the reputation value difference for high-reputation nodes is nearly negligible. This means that in NetTopoBFT, malicious nodes cannot directly attack core nodes based on their reputation scores. Here, we conduct further tests on the randomness of leader node elections to further validate the system’s security.

**Figure 5:**
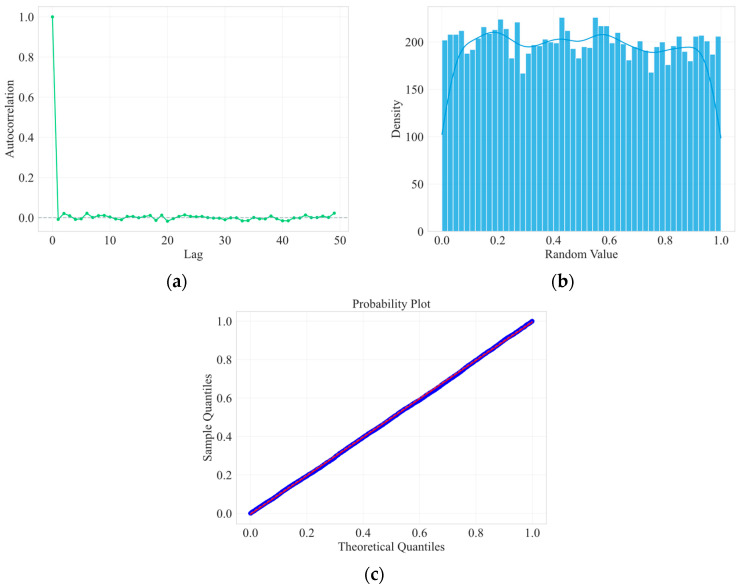
Probability distribution for leader node election. (**a**) shows the autocorrelation of the samples at different time lags; (**b**) shows the numerical distribution of the generated VRF random number samples; (**c**) is a quantile-quantile plot and tests the uniform distribution characteristics of the samples.

Figure 5 illustrates the unpredictability of leader node election using VRF from three perspectives. (a) is an autocorrelation plot, showing the autocorrelation of the samples at different time lags. As can be seen from the figure, all autocorrelation coefficients fall within a confidence interval close to 0, indicating no significant correlation. (b) shows the numerical distribution of the generated VRF random number samples. Both the bar chart and the KDE curve exhibit a flat shape, indicating uniform numerical distribution with no systematic bias. (c) is a quantile-quantile plot, which further tests the uniform distribution characteristics of the samples. As shown in the figure, all points are closely distributed around the reference line, indicating that the quantiles of the samples are highly consistent with the theoretical quantiles, demonstrating good uniformity. Therefore, malicious nodes find it difficult to launch targeted attacks on the leader node, ensuring the security of the leader node.

**Figure 6:**
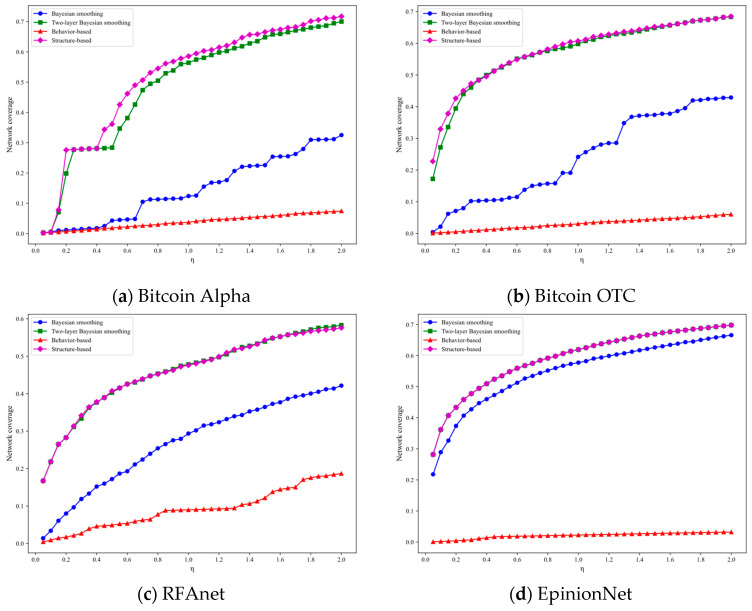
Consensus process network coverage.

Figure 6 illustrates the overall network coverage of NetTopoBFT’s consensus process across four datasets. Overall, when the number of nodes reaches a certain threshold, the network coverage of Figure 5a–c achieve network coverage exceeding the 66.7% coverage required for system security. It is foreseeable that the overall coverage will exceed 70%, encompassing nearly all trustworthy nodes in the network. The remaining 30% of nodes primarily include some edge nodes and nodes with negative weights in the network. These nodes, due to their special characteristics, are more susceptible to external attacks, thereby reducing the probability of malicious nodes participating in the consensus.

**Figure 7:**
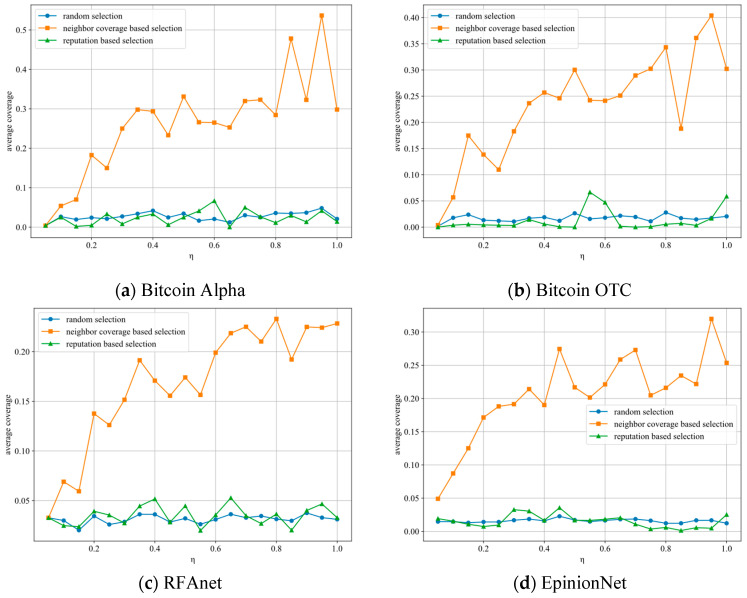
Backup node duplicate coverage rate.

When comparing the four algorithms vertically, we can further observe that simply using node behavioral reputation as the node reputation model (red curve) ultimately results in a network coverage rate that generally does not exceed 10%. which significantly compromises network security. Using Bayesian smoothing (blue curve) can to some extent ensure the overall network coverage rate, but only when the number of network nodes reaches ten thousand or more can a network coverage rate comparable to the other two types of algorithms be achieved. The algorithm proposed in this paper and the type of algorithm that establishes a node credibility model based on node structural characteristics both demonstrate comparable performance. When the number of consensus nodes reaches certain conditions, the overall network coverage can reach the threshold, ensuring the security of the consensus process. Additionally, Figure 7 further validates that the number of consensus nodes and the network’s topological structure are two important factors influencing network coverage. In practical applications, the appropriate number of consensus nodes y should be selected based on the network’s actual parameters to ensure the security of the entire system.

Notably, we also observe that the trends of the four curves in Figure 6c differ significantly from those in the remaining three figures, and none of the curves reach the 66.7% threshold standard. By combining the basic information of the RFAnet dataset in Table 3 with the experimental results in Figure 7c, we can conclude that the cause of the aforementioned results is determined by the network’s inherent structure.

Figure 7 illustrates the network redundancy coverage of candidate nodes for leader nodes in the NetTopoBFT algorithm and compares it with two existing methods: one based on descending credibility values and the other based on random selection of candidate nodes. The experiment was conducted η∈[0.05,2.05] with a step size of 0.05, taking into account the randomness of VRF elections. For different η, we conducted 100 experiments, taking the maximum network redundancy coverage from each experiment and calculating the average based on that. As shown in the figure, while the existing two algorithms can ensure that the replaced leader nodes have sufficient behavioral credibility, they cannot guarantee the equivalence of network coverage, which is equally important for consortium blockchains. The method proposed in this paper has a lower overall number of consensus nodes selected and a lower repetition coverage rate when η is small. However, as η increases, the repetition coverage rate can reach up to 56.53%, significantly ensuring the system’s security and the continuity of the consensus process. Although the repetition coverage rate of candidate nodes decreases overall as the network scale increases, it still has a significant advantage over existing algorithms.

## 6. Conclusions

Addressing the issue that existing BFT algorithms focus on performance optimization but lack systematic consideration of node structural characteristics and network coverage, this paper innovatively proposes a new network topology-aware Byzantine fault-tolerant algorithm, NetTopoBFT, tailored for applications such as supply chain management. The algorithm first leverages node structural characteristics to integrate weighted signed networks with consortium blockchains and proposes a two-layer Bayesian smoothing node evaluation model. It establishes a dual-dimensional evaluation mechanism combining ‘behavioral credibility’ and ‘structural importance’, ensuring high-quality node election results and reducing the likelihood of malicious nodes serving as consensus nodes. Second, it uses VRF to design a random election mechanism for leader nodes, ensuring the randomness of leader node elections and reducing the probability of attacks. Finally, it designs a backup node selection strategy based on network redundancy coverage, focusing on the stability and consistency of network message transmission. Compared to PBFT, NetTopoBFT has significant advantages in terms of communication complexity, fault tolerance, and dynamic update capabilities. Experimental results show that under the same conditions, this algorithm can better adapt to the overall environment of a consortium blockchain, balancing the structural characteristics of the consortium blockchain while ensuring system security, providing new insights for the design of consortium blockchain consensus mechanisms, and offering broad application prospects. This study currently focuses on the theoretical analysis and validation of the node selection model and security properties, and has not yet conducted large-scale throughput and latency performance testing. This represents our primary direction for future work to more comprehensively evaluate the practical utility of NetTopoBFT.

## Figures and Tables

**Table 1:** Classification of BFT Consensus Algorithms.

	Categories	Subcategories	Algorithms
PBFT	More efficient	Reducing message complexity	Zyzzyva [16], SBFT [17], Hotstuff [18]
		Ordering fairness	Pompe [19], Fairy [20]
		Reputation consensus	Prosecutor [21], PrestigeBFT [22]
		Multi-leader	MirBFT [23], ISS [24]
		DAG-based	DAG-Rider [25], Narwhal [26]
	More robust	Asynchronous and leaderless	DBFT [27], Honeybadger [28]
	More available	Defending various attacks	Timing attack: Prime [29]
			Performance attack: Aardvark [30]

**Table 2:** The key parameters used for the node evaluation model.

Symbol	Description
i,j	Node number
$R_{in},R_{out}$	The node’s original in-degree credibility and original out-degree credibility
$R_{in}\prime ,R_{out}\prime$	The nodes’ original smoothed in-degree credibility and out-degree credibility
deg_in(i)	Number of directed edges to a node i
deg_out(i)	Number of directed edges from node i
raw_r(i)	The nodes’ original smoothed reputation
global_avg	Global average reputation
R(i)	The global reputation of node i

**Table 3:** Comparison between different consensus mechanisms.

Consensus Mechanism	Communication Complexity	Byzantine Fault Tolerance	Scalability
PBFT	$O(n^{2})$	(n−1)/3	Low
Tendermint	$O(n^{2})$	(n−1)/3	Medium
DPoS	O(n)	-	High
Hotstuff	O(n)	(n−1)/3	High
Zyzzyva	O(n)	(n−1)/3	Medium
NetTopoBFT	$O(c^{2})$	≥(n−1)/3	High

**Table 4:** Key information of the four datasets.

Network	Soc-Sign-Bitcoinalpha	Soc-Sign-Bitcoinotc	RFAnet	EpinionNet
Vertices	3783	5881	9654	125,247
Edges	24,186	35,592	104,554	1,048,575
Average node degree	6.39	6.05	10.03	8.37
Median node degree	4	4	4	2
Maximum node degree	511	795	627	9734
Minimum node degree	1	1	1	1
Positive Edges	93%	89%	83.7%	95.42%

**Table 5:** Consensus Node Selection Results.

Proportion	Dataset	Index	Two-Layer Bayesian Smoothing	Bayesian Smoothing	Behavior-Based	Structure-Based
5%	Bitcoin Alpha	Q1	95.21	97.86	100	90.91
		$d\left(i\right)\geq \beta$	84.66	42.86	6.35	100
	Bitcoin OTC	Q1	89.00	97.55	98.16	84.54
		$d\left(i\right)\geq \beta$	98.64	55.44	5.1	100
	RFAnet	Q1	98.23	99.12	99.37	97.96
		$d\left(i\right)\geq \beta$	100	100	62.03	100
	EpinionNet	Q1	99.16	99.49	100	99.19
		$d\left(i\right)\geq \beta$	100	100	67.65	100
10%	Bitcoin Alpha	Q1	94.40	98.34	100	92.80
		$d\left(i\right)\geq \beta$	88.89	44.44	11.90	92.06
	Bitcoin OTC	Q1	91.95	97.52	87.11	87.11
		$d\left(i\right)\geq \beta$	98.3	54.59	11.39	91.84
	RFAnet	Q1	97.61	98.77	98.91	97.59
		$d\left(i\right)\geq \beta$	100	99.69	65.28	100
	EpinionNet	Q1	97.55	98.50	100	97.89
		$d\left(i\right)\geq \beta$	100	100	75.13	100
20%	Bitcoin Alpha	Q1	95.60	97.92	99.01	94.36
		$d\left(i\right)\geq \beta$	86.24	50.66	26.72	82.54
	Bitcoin OTC	Q1	92.01	97.42	89.44	89.44
		$d\left(i\right)\geq \beta$	84.78	53.32	21.17	77.47
	RFAnet	Q1	96.32	97.45	98.74	96.09
		$d\left(i\right)\geq \beta$	97.98	100	62.95	100
	EpinionNet	Q1	95.12	97.32	99.94	96.21
		$d\left(i\right)\geq \beta$	99.99	92.67	73.25	99.81

**Table 6:** The Varying Final Reputation Values Achieved by Malicious Nodes in BitcoinOTC.

	In-Degree Reputation	Out-Degree Reputation	Avg In-Degree Reputation	Avg Out-Degree Reputation	Degree Centrality	Total Reputation
1	50	50	10	10	5	2.39967
2	60	60	10	10	6	2.4421
3	70	70	10	10	7	2.46731
4	80	80	10	10	8	2.48170
5	90	90	10	10	9	2.48909
6	100	100	10	10	10	2.49190
7	200	200	10	10	20	2.44862
8	300	300	10	10	30	2.40087
9	500	500	10	10	50	2.34297
10	1000	1000	10	10	100	2.28443

## Data Availability

No new data were created or analyzed in this study. Data sharing is not applicable to this article.
